# How a Priority of Community Outreach and Engagement Is Changing Health Equity at Cancer Centers

**DOI:** 10.1089/heq.2021.29005.rtd

**Published:** 2021-04-21

**Authors:** Paul B. Jacobsen, Monica L. Baskin, Moon S. Chen, Roy S. Herbst, Christopher S. Lathan

**Affiliations:** ^1^ Associate Director, Health Care Delivery Research Program, Division of Cancer Control and Population Sciences, National Cancer Institute, Bethesda, Maryland, USA.; ^2^ Professor of Preventive Medicine, Associate Director for Community Outreach and Engagement, O'Neal Comprehensive Cancer Center, University of Alabama at Birmingham, Birmingham, Alabama, USA.; ^3^ Professor and Associate Director, Population Sciences & Community Outreach/Engagement, UC Davis Comprehensive Cancer Center, Sacramento, California, USA.; ^4^ Ensign Professor of Medicine (Medical Oncology) and Professor of Pharmacology, Chief of Medical Oncology, Yale Cancer Center and Smilow Cancer Hospital, New Haven, Connecticut, USA.; ^5^ Associate Cancer Center Director for Translational Research, Yale Cancer Center, New Haven, Connecticut, USA.; ^6^ Director for Cancer Care Equity, Dana-Farber Cancer Institute, Boston, Massachusetts, USA.; ^7^ Associate Medical Director, Dana-Farber Cancer Institute Network, Boston, Massachusetts, USA.; ^8^ Medical Director, Dana-Farber Cancer Institute, Brighton, Boston, Massachusetts, USA.; ^9^ Assistant Professor of Medicine, Harvard Medical School, Boston, Massachusetts, USA.

***Dr. Jacobsen*: Hello, everyone.**

**I would like to start our conversation by asking how the NCI's mandate that NCI-designated cancer centers address community outreach and engagement (COE) has impacted your institution from when it was first put in the Cancer Center Support Grant guidelines in 2012?**

**Dr. Herbst:** Outreach and engagement have been part of the NCI's requirements since 2012, but now these elements are actually being scored and evaluated. We talk about it, extensively both at our external advisory board meetings and in our weekly meetings here and we are really trying to have an impact in the community.

That has always been the goal of the cancer center and certainly of a NCI designated cancer center. But if you do not have resources, if you do not have plans, if you do not have collaborations, it is not going to work.

Because of this, at Yale, we made sure we had an associate director for COE. We were thrilled to be able to get Marcella Nunez-Smith to join the team in that capacity. We are doing work in the community with navigation, clinical trials, and prevention, and we have a grant from the Bristol Myers Squibb Foundation (BMSF) as well as support from the Dean's office to support these efforts, which was very helpful. These measures demonstrate what institutions want: ways to enhance diversity in clinical trials.

Also, we are making sure that every research program, of which we have seven programs in our cancer center, has someone who is a COE representative, who is working with this office to take their science to the community. The clinical disease-specific programs where the clinicians and translational researchers are based are also thinking about what they can do on this front.

This NCI mandate has really helped, at Yale we are working from both directions and have access to some resources, and certainly, being part of groups like this to get ideas from other centers has been huge for Yale.

**Dr. Baskin:** Our cancer center has certainly had a long history of conducting outreach and, in fact, this year we are celebrating 25 years of outreach and engagement in Alabama. However, since the implementation of the Cancer Center Support Grant (CCSG) requirement, outreach and engagement have moved from being what you might think of as just a “nice gesture” to being something that is showcased front and center.

Outreach and engagement are now part of all of our conversations and strategic leadership meetings. We are often talking about outreach and engagement and how we can better serve the needs of our catchment area. I think it has really been critical to have the NCI guidance, particularly having a major contribution to the CCSG application scoring, which definitely gets people's attention. That has been really critical in making these activities a focal point of what we do and ensuring that resources are appropriately allocated to this work.

**Dr. Lathan:** I would like to echo what Dr. Herbst and Dr. Baskin described. It is a similar situation at the Dana-Farber/Harvard Cancer Center. We have always been interested in and dedicated to engagement. I think that to be really specific, the NCI putting teeth into this really changed outreach and engagement from what used to be considered “nice to have” into a “must have.” This move was really making the steps toward accountability and as you heard from Dr. Herbst and Dr. Baskin, without having a budget, much of this work has been built on the sweat equity of the people (researchers, community-based educators, and clinical teams) who are really interested in this.

So, although I think it was seen as being important, the truth is that if it were to be truly important, it would have needed a budget to be truly effective. It should also be noted that even despite not having a budget, some institutions and people have done incredible things, but now that there is a scoring element, there is some accountability to it.

We have an incredible group of people who are running our institution's community engagement and now suddenly there is a budget, a robust budget, probably not as robust as I would like but much more than it used to be. Similarly, there is now a push to integrate this beyond just a few like-minded thinkers and into the deep recesses of the research, including clinical, health services research, and also clinical trials. This growth has been fantastic to observe.

**Dr. Chen, Jr.:** Having just experienced the site visit this week (February 2, 2021), I am very fresh about everything, and let me just tell you that having the NCI elevate the role of COE has been tremendous. I am now the extension of the director in making sure that COE is an integral part of fulfilling the mission and aims of the UC Davis Comprehensive Cancer Center.

In fact, I attend all the research meetings of all the five cancer center research programs that we have. I have worked with each of the program co-leaders to make sure that there is a plan and a product to make sure that their current and future portfolio addresses the catchment area's cancer burden.

In fact, the associate director in clinical research is telling us now that the NCI's emphasis is not just in the number of people being recruited to clinical trials, but also in the explicit expectation that those patients come from the catchment area and that they are from diverse populations. A COE faculty member (Julie Dang, PhD, MPH) is on the scientific review committee to make sure that diverse populations are included in all the new clinical trials and that those trials are intentionally addressing the cancer burden.

We (COEs) are everywhere, to the point where the director has lightened my load by saying that I no longer need to have oversight of population sciences (a new associate director for population sciences has been appointed). Because we have got so much to do in COE that I am just going to be the associate director for COE exclusively.

The role of the COE is to nurture relationships with community. In the past 3 years, we have been very successful in being funded for at least $9 million worth of extramural grants.

At our cancer center, COE is the public face to the community.

I have been told the second most important determinant of the overall CCSG score for evaluating NCI cancer centers. This is welcomed as this requires COE to distinguish itself in the careful characterization of the cancer burden in our catchment area, that we are harnessing the full spectrum of cancer research: basic, clinical, translational, and population sciences to bear on reducing the cancer burden in our catchment area as well as to collaborate with other university and nonuniversity entities in this mission.

I really like that the emphasis on the catchment area means that no cancer center is going to be the same, but rather we have a distinct responsibility to the people who are served by the cancer center either because they seek care here but also where we conduct outreach and engagement to extend the benefits of research to them. For our catchment area, we do that by seeking the wisdom of our community advisory board (CAB) who helps prioritize our work and who also evaluates our outcomes. It is very exciting and gratifying to me to see the elevation of the importance of this.

***Dr. Jacobsen*: That is very gratifying for me to hear because it is very consistent with the intentions of my colleagues at NCI who drafted that language. Their goal was to make sure that NCI-designated cancer centers were responsive to their local communities in the ways that we are going to talk about.**

**It feels like our convening session was held in a different era when people used to get on airplanes, stay in hotels, and meet in large rooms without masks. Many other events in 2020, including the serious dialogue that has occurred in the United States about systemic racism in response to the murder of George Floyd and others, also make it seem like the convening took place much longer ago than it actually did. It is a different world now, and I am wondering whether the roundtable participants would be able to reflect on how your cancer center has evolved in response to the events of 2020.**

**Dr. Baskin:** Our catchment area for the O'Neal Comprehensive Cancer Center is the state of Alabama. This area includes >26% of Black Americans. So certainly, this is a topic that is very relevant to the people we serve.

Alabama is also a state with a very long and ugly history of systemic racism, which is clearly a root cause of the killings that happened and received national attention last year. This is a community that has long grappled with these kinds of issues. The people in these communities have very vivid memories if not recollections of stories about family members having been lynched, so these issues are very much top of mind.

As a result, we have always been sensitive to issues related to systemic racism, particularly our work in largely Black rural communities. Part of our success, our use of a model for outreach and engagement where we employ local staff and volunteers to be the frontline team members to disseminate messages about cancer prevention and control and the higher burden of cancer among Black and rural residents.

Using this local connection, over the past several months we have responded to the major events of 2020 by constantly checking back in with community members to see how things are going and really get a sense of how they have been impacted. From there, we have looked to identify and communicate local resources to address these needs (e.g., COVID-19 testing, food assistance, and mental health care).

Frankly, we have also had to be very careful about what messages we were putting out. First and foremost, we made sure we had accurate information about what was happening with the pandemic, and how people could keep themselves safe and reduce risk. We were more cautious about promoting testing and vaccination, because oftentimes in the communities that we serve, testing and vaccinations were not widely available. We had to balance a push for doing these things when we knew that those resources were not available.

Part of what we did was identify where testing locations were, and to try to match that up with our knowledge of local interest. We also made sure that we continued our message about the need for cancer prevention and care despite COVID-19.

We collaborated, for example, with our State Department of Public Health to get co-branded messages out there about staying safe while not neglecting in-office visits for care. Then, in our local communities, we also made sure that there were appropriate resources available such as tablet computers and WiFi access, as many of those appointments then move from in-person to telemedicine and many people in our community just did not have access to the high-speed internet or even video capability.

So, we sprang into action, and I think we have been able to be very responsive because we have had this infrastructure for years. But again, we have had to balance out how much we are moving forward, ensuring that we were always being very upfront, providing factual information and resources to people to get care.

**Dr. Lathan:** I have come to start describing time as pre-Floyd and post-Floyd because Mr. Floyd was not the first Black American to be killed as a victim of structural racism. But his death served as an inflection point in the way that it impacted America, which has been really dramatic.

Before the death of George Floyd, I never used the phrase “structural racism” in my talks. And, as a physician, I did not talk about social justice, not because I did not believe it was important, but rather because it really was not quite acceptable and it made people uncomfortable. We talked about the impact of social and demographic factors, but we were not specifically calling out structural racism. We in the cancer world were not specifically talking about social justice as part of our mission. This change in tone has allowed us to really focus on how to engage with communities while keeping those concepts in mind. All of the incredible work that we have all done, whether it is disease based or health service based, we can now really call out the role of social issues and structural racism being a part of this to enact impactful change. Specifically, I think if we look at the COVID-19 experience, regarding the distribution of vaccines and the roll out and utilization of telehealth, these are areas where we can start to make change. I have said this for years, as have other like-minded people, that engaging with our communities and listening to them are important.

It is not that people would not listen before, but they would say, “Ah, there's some competing priority that will not allow us to do what it is that you would like to do.” Now, when you say it, you still have to push for things to get done, but other people are listening.

I think leadership is able to try enact some of these processes to really rectify the issues, and recognize that this had a palpable effect on our Black, Brown, working poor, and immigrant communities. I do think it made an incredible difference and created a moment in which we could really try to make substantial differences in the way that we deliver care and engage with communities.

**Dr. Chen, Jr.:** One of the first things we did was to characterize the demographics of our catchment area population. Our demographic profile differs from the United States as a whole. The majority of our catchment area population are racial/ethnic minorities (57%). Proportionally, we have more of every federally defined racial/ethnic population with the exception of African Americans (8% vs. 14% nationwide). Specifically, our catchment area is 29% Hispanic versus 17% for the United States; Asian Pacific Islanders, 1% versus 7% nationwide; Native Americans, 3% versus 2% nationwide.

So we recruited the highest ranking African American cancer surgeon, Dr. David Cooke, in our cancer center, and he has taken a volunteer leadership role in the African American initiative, focusing first on increasing lung cancer screening for eligible African Americans. That has been the leading way in which we are structuring this effort. We also have faculty leads and COE staff for each of the other racial/ethnic populations as well as one for rural populations.

After COVID-19 became a reality, and after the other social injustice incidents, we have only intensified our focus on African American communities, although access has been delayed, in part, because of COVID-19. But our commitment and emphasis remain the same in that we want to prioritize focus on lung cancer screening for all eligible African Americans.

The other thing we are fortunate to have is a separate P-30 supplement on COVID-19. Dr. Cooke and I are co-leaders in this, and that he has recognized one of the challenges we need to do, and he is taking leadership on this to figure out how we can reduce the barriers to cancer care through telehealth and telemedicine.

**Dr. Herbst:** The events of 2020 certainly struck everyone, no matter who you are. I look out in the streets of New Haven beside me and see how people have been coming together: students and faculty alike, patient's candlelight ceremonies, and moments of observance, especially in a city like mine, New Haven, which is quite diverse.

And then of course we are focusing on COVID-19. We pivoted a little bit and placed much of our attention on COVID-19 during this time. Look at who was in the hospital/ICU with COVID-19. One quickly noticed that the majority of those being hospitalized were people from low socioeconomic backgrounds, showcasing the structural disparities that we talk about. You could see it on every ward. Those in the ICU who were intubated were largely from minority groups or the elderly.

COVID-19 really pointed out the disparities that exist in health care. At Yale we have a group led by Dr. Albert Ko, who is an epidemiologist, and Dr. Marcella Nunez-Smith, who looked at data from 11,000 patients in a database of Yale New Haven health. The patients include people from the Interstate-95 corridor all the way from Greenwich up to Boston, and then a little bit west of Hartford. Fifty percent to 60% of the patients who died of COVID-19 in this group were either African American or LatinX. That really told us that there are disparities in health care, and we reached out to help this community using resources from our existing and new grants from the BMSF. We used some of our already existing patient navigators to deliver masks and work to make the community more comfortable with vaccines. We did our Yale vaccine trials in those communities, and we used some of the cancer center resources to help conduct clinical studies.

Importantly, through all this we are reaching out more to Yale researchers who are now working with the cancer center in the community. We all realized through this experience that we really have to reach out more for cancer care as well. It was an eye-opening experience, and now we are using the same trust that we have hopefully built and additional resources to go out to the community and take care of our catchment area and provide more cessation and prevention methods for smoking and provide more screening. And then of course, we are making sure all people have access to care. Certainly, for a state like Connecticut, we really wish of course that the pandemic had not happened, but it got us accelerated in some of the things that we need to do to fight cancer.

***Dr. Jacobsen*: An important component of COE is to make sure that cancer centers understand they have a responsibility for the cancer burden in their catchment area and to make sure that the work of the cancer center extends to the community. But another important component of COE is that the community's needs influence the work of the cancer center.**

**I want to ask each of you to reflect on how COE may have brought a deeper understanding of the community's needs and its desired outcomes to your cancer center, and possibly influenced the research agenda.**

**Dr. Lathan:** It is supposed to be bidirectional, right? But as we talk about structural racism, we have just talked about the inequity that happened in that relationship. One part of this is a community that is vulnerable in many ways, and on the other side is a cancer center.

So generally, this conversation had been unidirectional. The previous approach was to go to the community and advise them about new trials and asking them to join.

I think what has happened is there has been more listening, and I think we could all use more of that. Although it does not take away that inequity and power that we just talked about, if you look at how cancer centers have engaged around COVID-19, this is a good indicator that I think they are listening to what the community needs, not necessarily only what the cancer center needs.

I think that is hard for every cancer center, not just mine. I think there has been a little bit more of that bidirectionality, but I still think you need to have sustained listening, and we really want to make sure that this bidirectionality is the seat at the table for the community is not just on your board that you come to, but also maybe participants sit on your clinical trial task force and actually push some things forward, perhaps making the cancer center think about things that they are less comfortable with and hearing some of these innovations as opposed to the other way around.

I think we will be seeing that going forward. That is where I would say that this should be bidirectional, we have been seeing evidence of this, our cancer center and other cancer centers have been doing this around COVID-19. I hope it sustains and continues.

**Dr. Baskin:** I definitely echo some of the same kinds of comments that Dr. Lathan made. I think certainly an advantage of having this focus area is it is requiring us to sit down with members of our catchment area, again, to see what their needs are. We have also been able to get multiple supplemental funds from NCI to do assessments of our catchment, which have been really critical.

We have also been focusing on our rural areas, and that has given us a deeper dive into what those needs are at the local level to start to help and be integrated into our overall strategic plan. As Dr. Lathan mentioned, really thinking about those long-term sustainable issues is important.

Part of what we were able to do was to add on a couple of other areas of focus in terms of certain cancers that we have never really focused on before, because we looked at those data and we talked to community members who asked that we do more. For example, we had not had a great outreach around prostate cancer for a number of years. As we looked at the epidemiology data, and we spoke with many African American men across the state, they said this is really a major issue in our communities, but “you (our cancer center) are silent on this.” So, over the past 2 years we started ramping up our outreach, and we had to figure out what the message would be. That was a delicate balancing act as the recommendations around prostate cancer screening were not consistent, making it challenging to offer a straightforward prevention message.

We got a little bit of a window with the current guidelines recommended shared decision making. We took that and were able to run forward to address community concerns. So, I think it is really important to bring them to the table.

And we have also integrated COE into senior leadership. We have a formal CAB with more than 22 members representing individuals from across our state. On a quarterly basis, they meet with program leaders to talk about what the community's interests are, but this also allows our program leaders to talk about what is happening in the program. And there is interaction back and forth about what is working and what is not working.

We review and provide support to many of our investigators doing research, and we have a community member who reviews that research, the methodologies that are out there, and give input on that.

We have two cochairs of that board, and they meet on a regular basis with the cancer center director. Being at the table being in the room where decisions are being made, that is something that certainly was not happening before.

**Dr. Herbst:** We are seeing the same things. Our cancer center has expanded, but we realize the mostly suburban sites that we have opened really do not fully understand these issues and deal with the disparities, and we just look to our inner city, and realize we had to listen and find out what the issues and problems were. We have a CAB too, which we have enhanced, and call our “cultural ambassadors,” and we brought people in, we listened, some of our physicians (myself included) have been at some local radio shows that reached out to the community, some of the reverends and pastors and so forth. And we really heard that there remain issues with trust and access.

We have patients with lung, prostate, colon, breast, and bowel cancers at increased numbers in our community compared with the national average, so we are trying to reach out with screening and prevention. I think we are doing a bit better there, such as by putting more resources into mobile health care services, like our community health care van, which supported reductions in community transmission of COVID-19 by distributing personal protective equipment and educational materials as well as telehealth coordination and postpartum care closer to home. We now have a mammography van for breast cancer screening, but also they are trying to get clinical trials and get people at least just to come in, but then encouraging people through navigators to think about clinical trials.

If you have a clinical trial, at least you know you are thinking about the standard of care and maybe even doing a bit better. So, we have really pushed that. The diverse training programs that are now in place through the BMSF aim to get more diversity in our workforce. If you do not have a diverse workforce that represents the community you treat, you will not be as successful, and you will not build the trust that we want and need. We have been very much trying to diversify our recruitments, and we are very happy that there are funding sources that try to support young investigators who want to learn how to do clinical trials and research with diverse populations. This has been a time of great thought and building and a lot of this has been stimulated through these NCI mechanisms.

**Dr. Chen, Jr.:** At the UC Davis Comprehensive Cancer Center, we also agree on the prominence of COE in fulfilling our cancer center's mission. This year (2020–2021) is the first year that we have applied for or been evaluated on COE. And I can say, I am very proud of our cancer center director personally involved in COE and supporting COE even before CCSG funding.

For instance, our director covered the difference in the indirect rates from the BMSF funding. He viewed it as a great investment, so we are very appreciative of that, and also the value that this stimulated for us to collaborating with the largest federally qualified community health center serving Asian Americans in Sacramento County to mitigate the unnecessary burden of hepatitis B and its linkage to liver cancer.

The second contribution that our cancer center made in 2018 was to invest in developing a CAB. As a consequence, through our years with the CAB, we are really proud of the fact that these CAB members reflect all parts of the catchment area, all the counties, different professions, and are balanced in terms of gender as well as racial and ethnic distribution, and that they were those when we first met with them who said we needed a strategic plan for COE.

So, we prepared a county-based strategic plan, and CAB comments on what needs to be done. And then they have further provided us with prioritization given what we know about our catchment area and cancer burden. We were advised of the four areas to focus on: tobacco, HPV, hepatitis B, and colorectal cancer screening.

They wrote an evaluation of our program to the director and advised on what to adopt as the priorities and so that the director in our most recent CCSG application included that as what we would do and described how we would have a program to back that. For example, we were able to get funding for three out of those four areas, had to scour the internet to find that there was a funding opportunity for colorectal cancer screening, which we adapted. So I cannot speak enough about the importance of our COE and how active they are, and we have invited them to participate and provide guidance on a particular clinical trial attempt that we are doing. This particular clinical trial in the past has not attracted minorities except for one in the whole batch.

So we invited them and they talked with our radiology scientist about this, and they were very receptive. So now we have a videotape of the importance from the point of view of racial ethnic minorities on the value of participating in cancer imaging, in our total PET scan. We are launching that very soon.

***Dr. Jacobsen*: You are all familiar with the recommendations that came out of the convening session that are captured in the publication. Which one or ones are your cancer center focusing on to advance its work in health equity and community outreach engagement?**

**Dr. Chen, Jr.:** The idea of using the evaluation criteria, I think it is an area that we need to focus on, and I think this is an area that we are needing with various metrics. And we need to have better metrics of our COE impact, because COE does not, we have been told, conduct research, but yet, we believe intra- and interprogrammatic publications would be an important metric and is analogous to what the CCSG program does.

Thus, just as CCSG research programs have metrics on intraprogrammatic, interprogrammatic, and multi-institutional collaborations, we at our COE measure ourselves in terms of the extent to which our own cancer center members (intraprogrammatic) and our community partners (interprogrammatic) coauthor the articles that are generated from our COE-initiated studies. We count the number of external awards in which a portion of the budget goes to our external community partners. And we count the number of external entities that contribute to the publications of our findings. These are tangible evaluation metrics we use in assessing the extent to which the community is part of our COE and our COE is part of our community.

**Dr. Herbst:** I picked two from the list. First, we were trying to do a COE pilot—as I mentioned earlier—pairing nearly every program with a COE representative so, we got each program to look at the research and—through either using patient samples or outcomes work—to bring some implementation to the community, some sort of screening method to something related to that research program.

And then the second thing we were really focused on here is clinical trials. We brought in the practice of Dr. Andrea Silber, who has been a practicing oncologist in the community for many years, and has put together a number of grants and programs to provide navigators. Then we have bolstered that with other support, some from the BMSF, to really try to get out to the community and bring people in for the different clinical trials.

Our minority accrual on trials has been low, in the 5% to 10% range, and we are trying to improve that and provide access. That is easily quantifiable metric. So we are working hard on that, trying to take advantage of the science and unique things that can take advantage of our faculty and their expertise.

**Dr. Lathan:** I think similarly, picking two areas. One, similar to Dr. Herbst's clinical trials, I think some of those efforts, specifically, are looking at expanding our partners whom we work with, looking at our networks, and then even thinking bigger about how our whole infrastructure of clinical trials work. The guidelines are exclusionary, so we are focusing on really breaking down the whole process.

The other thing, which is something that has been near and dear to my heart, is expanding access and clinical pathways. And you can do that many different ways. It is all about relationship building, whether that is using navigators, or building relationships with federally qualified health centers, which is an area that our cancer center has been interested in.

Another example might be collaborations with our other academic centers that might be treating more patients of color and more immigrant patients and building the clinical trials infrastructure with them.

This is not just about researchers wanting more people of color to come to our trials, which of course we do, but the focus is on building the infrastructure where patients can go and get treated wherever they want. Access to care, as well as looking at clinical trials, is really where we have been broadening our programs.

**Dr. Baskin:** I think I will continue that theme. I think of the two things that come to my mind, one of them is certainly trying to bring in our basic sciences to better understand what COE is and have that integration in their work. We also have a pilot program going on. We have trainees learning about community and culture who are interacting with individuals in our catchment area. They are given an opportunity to apply for pilot funds to do research that is driven by what they have learned by interacting in the community, so making sure that basic science is integrated with COE.

We were also focused on ensuring the highest quality of care for everyone in our catchment area. We know that clinical trials can be one of those areas in which people can get high-quality care, so we are really focused on making sure that that access is equitable for everyone. Part of that is making sure people are aware of the trials that exist and reducing structural barriers to people getting enrolled in the trials, including overcoming related financial challenges (lodging and transportation) all the way through potential biases from the research team about who they are engaging for those trials. Those are the two things that I would say we are actively working on.

***Dr. Jacobsen*: I just want to add that, on the NCI's part, we have engaged a number of activities to support COE. Perhaps the most tangible example is that we issued a request for administrative supplements to cancer center support grants to build capacity related to COE. We offered centers the option of using those funds to help build relationships between their basic science programs, as Dr. Baskin just mentioned, and their community partners, or to use the funds to adapt, implement, and evaluate existing evidence-based interventions in collaboration with community stakeholders. We issued 23 awards to cancer centers through those administrative supplements.**

**Here is my last question. How can cancer centers work together to take actions that will address disparities in access to care, quality of care, and health outcomes?**

**Dr. Herbst:** I am going to suggest public private partnerships, like the lung cancer master protocol we are doing with the Foundation for the National Institutes of Health (FNIH) that is open at 750 sites throughout the United States.

That trial provides profiling and drugs based on molecular markers. Those 700+ sites are in very diverse areas. We create public–private partnerships, bringing together pharma to supply drug and/or financial support and the FNIH with its access to the community through NCI Community Oncology Research Program and various other including multiple NCI cancer centers and cooperative groups—all working together.

That type of mechanism is bringing science, is bringing profiling to diverse populations, and is bringing drugs to populations with funding that normally would not have it. So I think that is a mechanism we should explore more. We were only going to make a difference here if we all collaborate. We want everyone to have access to precision medicine, and the NCI centers. These are the types of things we need to do more of.

**Dr. Baskin:** I will continue the theme of collaboration. I think one of the things that would certainly be helpful is to continue the various learning communities with individuals who are focused on COE. This group should continue to have a forum by which we come together, because some of the solutions that are in certain areas may, with a little tweaking, work in other places.

We do not always have to be creating something that is new or specific to only one center. We can learn from one another, and I think those learning communities provide a place where we can share that knowledge and move forward. I think a perfect example is another supplement that we were able to get around COVID-19, for example. Dr. Chen is on that with us. We have 17 cancer centers that are focused on collecting information about the impact of COVID-19 and how that is impacting cancer prevention and care.

So we were learning from one another, and we were using a shared group of metrics that we are going to evaluate and I think that is helpful. Lastly, many of us have talked about community at the workshop, and how this work is mentally and physically challenging. Simply having a place to go and have conversations with like-minded individuals, talk about the challenges, and learn from others and have support from others are critical for us to be able to come up with the solutions to address the disparities.

**Dr. Lathan:** Dr. Baskin's words really resonate with me. We can impact policy when we bring all of these cancer centers together.

We can talk about social justice now. We can talk about structural racism, we can hear similar problems all the way through and now we know to go back to NCI to go back to Medicaid, Medicare, and other payers, and acknowledge that these disparities are a universal problem that need a policy change in addition to all of us doing great science and sharing ideas.

I think the collective pooling and sharing of ideas are important. As Dr. Baskin says, we do not have to reinvent the wheel every time, because there has been a lot of wheel reinvention in COEs, and we want to make sure we do not have to do that because we can stand on the shoulders of those before us.

**Dr. Chen, Jr.:** I am really appreciative of the BMSF convening us in 2019. It was tremendous to have that opportunity for sharing and I know that before that there was a COE-specific meeting in Minnesota and another one coming up in LA later on this year. I think these provide a tremendous opportunity to share best practices and lessons learned. I think that is the way to do it. That way we kind of cross-pollinate each other with great ideas. I have benefited from that and I believe that it can only get better.

***Dr. Jacobsen*: Thank you. I am glad you mentioned that meeting. It is a virtual meeting hosted by Cedars-Sinai Medical Center and Stanford University at the end of April 2021, Thank you all for participating in this excellent discussion.**

